# SOX9 inactivation affects the proliferation and differentiation of human lung organoids

**DOI:** 10.1186/s13287-021-02422-6

**Published:** 2021-06-10

**Authors:** Lian Li, Jianqi Feng, Shanshan Zhao, Zhili Rong, Ying Lin

**Affiliations:** 1 State Key Laboratory of Organ Failure Research, National Clinical Research Center of Kidney Disease, Key Laboratory of Organ Failure Research (Ministry of Education), Cancer Research Institute, School of Basic Medical Sciences, Southern Medical University, Guangzhou, 510515 China; 2 Dermatology Hospital, Southern Medical University, Guangzhou, 510091 China; 3 Bioland Laboratory (Guangzhou Regenerative Medicine and Health Guangdong Laboratory), Guangzhou, 510005 China

**Keywords:** Lung organoids, SOX9, CRISPR/Cas9, Proliferation, Differentiation

## Abstract

**Background:**

The regulation of the transcription factor sex-determining region Y-box transcription factor 9 (SOX9) in lung development has been described in mouse, but the same principles apply to human lung development is unknown due to a lack of appropriate experimental approaches and models.

**Methods:**

Here, we used gene editing technology to inactivate SOX9 in human embryonic stem cells that were then induced to differentiate into lung organoids to investigate the role of SOX9 in human lung epithelium development.

**Results:**

Complete knockout of the transactivation domain of SOX9 by gene editing resulted in indels in both alleles of *SOX9*. SOX9^−/−^ hESCs could be induced to differentiate into lung progenitor organoids. In vitro long-term expansion showed that *SOX9* inactivation did not affect the differentiation of pulmonary epithelial cells, but promoted apoptosis and reduced proliferative capacity in the organoids. When lung progenitor organoids were transplanted under the kidney capsule of immunodeficient mice, expression of the club cell marker secretoglobin family 1A member 1 (SCGB1A1) was detected in SOX9^−/−^ transplants but was absent in wild-type (WT) transplants. The maturation of goblet cells was also affected by SOX9 inactivation, as evidenced by the presence of mucin 5 AC (MUC5AC) in the cytoplasm of SOX9^−/−^ grafts as compared to WT grafts in which most MUC5AC was secreted into the lumen. In vivo lung orthotopic transplantations showed that *SOX9* inactivation had a limited effect on the differentiation of alveolar cells and lung regeneration in injured mice.

**Conclusions:**

SOX9 modulates the proliferative capacity of lung epithelium but is not an indispensable transcription factor in the regulation of human lung epithelium development.

**Supplementary Information:**

The online version contains supplementary material available at 10.1186/s13287-021-02422-6.

## Background

The lung is one of the most important organs of the human body as it enables efficient gas exchange between air and blood. In mammals, this function requires a complex tissue structure consisting of multiple cell types that is connected to other systems such as the cardiovascular system. The anatomic and functional complexity is the product of a stepwise developmental process involving precisely controlled proliferation, differentiation, and apoptosis along with multicellular self-organization and -patterning. The endoderm passes through embryonic, pseudoglandular, canalicular, saccular, and alveolarization stages to achieve the highly branched construction of mature lungs [1].

Early lung progenitors (LPs) in mice are characterized by the expression of the transcription factor NKX2.1 from proximal to distal lung epithelium, which is followed by proximal sex-determining region Y-box transcription factor 2 (Sox2) and distal Sox9 and inhibitor of DNA-binding 2 (Id2) expression [1]. In contrast, in the developing human lung, pseudoglandular tip progenitors are Sox2^+^/Sox9^+^ and become Sox2^−^/Sox9^+^ during the canalicular stage [2].

The transcription factor Sox9 was first investigated in the context of campomelic dysplasia (CD), a disease related to defective chondrogenesis and XY sex reversal [3]. During lung development, Sox9 is a marker of distal LPs and cartilage formation [1, 4]. A study using SP-CrtTA/tetOCre/Sox9^flox/flox^ mice reported normal lung morphology, differentiation, and tissue repair following oxygen-induced lung injury after Sox9 inactivation [5]. However, it was later shown that Sox9 is essential for proper branching morphogenesis and lung epithelium development. Conditional Sox9 ablation in the lung epithelium using Shh-Cre/Sox9^flox/flox^ mice caused a lethal branching defect in embryos, inappropriate epithelial cell proliferation and differentiation, and multiple cellular lesions [6]. Mesenchyme-specific knockout of Sox9 in Tbx4-rtTA/tetOCre/Sox9^flox/flox^ mice resulted in cartilage ring defects and aberrant differentiation of the airway epithelium [4, 7]. These conflicting findings on the role of Sox9 in lung development may be attributable to the different genetic backgrounds of the mice that were used in the studies [8], which reflects the limitation of using a mouse model to study development processes. Moreover, because of species differences, the progression of human lung development and disease cannot be easily or accurately modeled using animal models. As such, there is a lack of information regarding the regulation of SOX9 during human lung development.

Organoids are stem cell-derived 3-dimensional (3D) structures that contain multiple self-assembled cell types supported by extracellular matrix [9]. The spatial arrangement and cell–cell interactions of organoids mimic those of the native organ, making them a powerful tool to study human developmental and disease processes [9, 10]. In this study, we used lung organoids derived from SOX9^−/−^ human embryonic stem cells (hESCs) generated using gene editing technology to study the role of SOX9 during human lung development.

## Methods

### Maintenance of hESCs

The H9 hESC line was obtained from WiCell Research Institute. Stem cells were maintained in mTeSR1 medium (STEMCELL Technologies, Vancouver, BC, Canada) on plates coated with Matrigel (BD Biosciences, Franklin Lakes, NJ, USA; cat. no. 354277) with the medium changed daily. Cells were passaged by digestion with TrypLE Express (Gibco, Grand Island, NY, USA) and reseeding at a concentration of 1:10–1:15.

### CRISPR design and targeted mutagenesis

A *SOX9*-null H9 line was generated using the CRISPR/Cas9 method. Briefly, gRNAs targeting exon 3 of the *SOX9* gene were cloned into a vector containing the puromycin resistance gene (gRNA1, 5′-GGGCTGTAGGCGATCTGTTGGGG-3′; gRNA2, 5′-TCCTACTACAGCCACGCGGCAGG-3′). gRNAs and Cas9 plasmid DNAs were combined and transfected into H9 cells. Positive clones were obtained by puromycin selection and seeded at a limiting dilution for subcloning. Individual colonies were isolated and expanded. For genotyping, genomic DNA was isolated from cells to screen for SOX9 deletion by sequencing a PCR-amplified fragment spanning the 2 gRNA sites.

### Induction and passaging of airway and alveolar organoids

Stepwise differentiation of hESCs was carried out as previously described [11, 12], with some modifications. Briefly, to induce definitive endoderm (DE), hESCs (~ 90% confluence) were cultured in 24-well tissue culture dishes for 3 days in RPMI1640 medium containing 100 ng/ml activin A (R&D Systems, Minneapolis, MN, USA; cat. no. 338-AC-050) and 2 μM CHIR99021 (Tocris Bioscience, Bristol, UK; cat. no. 4423-10MG). From days 4–7, the medium was replaced with Advanced DMEM/F12 (Life Technologies, Carlsbad, CA, USA; cat. no. 12634010) supplemented with 200 ng/ml Noggin (R&D Systems; cat. no. 6057-NG-100), 500 ng/ml fibroblast growth factor 4 (FGF4) (Peprotech, Rocky Hill, NJ, USA; cat. no. 100-31-1MG), 2 μM CHIR99021, and 10 μM SB431542 (Tocris Bioscience; cat. no. 1614-10MG) to generate anterior foregut endoderm (AFE). On day 8, cells were embedded in Matrigel (BD Biosciences; cat. no. 356237) to initiate the 3D culture. “Ventralized” anterior foregut endoderm (VAFE) was generated by culturing cells in Dulbecco’s Modified Eagle’s Medium (DMEM)/F12 (Life Technologies; cat. no. 11320033) with 20 ng/ml human bone morphogenetic protein 4 (BMP4) (R&D Systems; cat. no. PRD314-10), 0.5 μM all-trans retinoic acid (Sigma-Aldrich, St. Louis, MO, USA; cat. no. R2625), 3.5 μM CHIR99021, 1% Glutamax (Gibco; cat. no. 35050061), and 2% B27 supplement (Life Technologies; cat. no. 17504044) from days 8–14. For LP induction, VAFE-enriched cells were cultured in DMEM/F12 supplemented with 3 μM CHIR99021, 10 ng/ml human FGF10 (R&D Systems; cat. no. 345-FG-025), 10 ng/ml human keratinocyte growth factor (KGF) (Novoprotein, Shanghai, China; cat. no. CM88), and 20 μM DAPT (Sigma-Aldrich; cat. no. D5942) from days 15–21. To generate airway organoids, the cells were incubated in Ham’s F12 (Gibco; cat. no. 21127022) with 50 nM dexamethasone (Sigma-Aldrich; cat. no. D4902), 100 nM 8-Br-cAMP (Biolog Life Science Institute, Bremen, Germany; cat. no. B007-500), 100 nM 3-isobutyl-1-methylxanthine (Wako, Osaka, Japan; cat. no. 095-03413), 10 ng/ml KGF, 1% B-27 supplement, 0.25% bovine serum albumin (BSA) (Sigma-Aldrich; cat. no. A1470), 15 mM HEPES (Sigma-Aldrich; cat. no. H0887), 0.8 mM CaCl_2_ (Sigma-Aldrich; cat. no. C3881), and 0.1% ITS premix (Corning, NY, USA; cat. no. 354351) starting from day 21. For human alveolar organoid induction, the above-described human airway organoid medium was supplemented with 3 μM CHIR99021 and 10 μM SB431542. To passage organoids in a cluster, cell aggregates were mixed with fresh precooled Matrigel and placed in a 12-well cell culture plate. After incubation at 37 °C for 20 min, 1.5 ml of medium was added to the plates, with medium replacement every 3 days. For single-cell passaging, organoids were incubated in 0.1% trypsin-EDTA (0.25% trypsin-EDTA [Gibco] diluted with Dulbecco’s phosphate-buffered saline [DPBS]) to obtain single cells that were passaged in fresh Matrigel.

### Real-time qPCR

Total RNA was extracted using TRIzol reagent (Molecular Research Center, Cincinnati, OH, USA; cat. no. TR1187), and 1 μg was reverse transcribed using the Evo M-MLV RT Kit (Accurate Biology; cat. no. AG11711). The cDNA was diluted and used as the template for qPCR, which was performed with the SYBR Green Premix Pro Taq HS qPCR Kit (Accurate Biology; cat. no. AG11701). The glyceraldehyde 3-phosphate dehydrogenase (*GAPDH*) gene was used as the internal control. Three biological replicates of each sample were prepared, and data are presented as the mean ± SD. Primers used for amplification are listed in Additional file 1: Table S1.

### Immunofluorescence and histological analyses

For immunofluorescence labeling, organoids or lung tissue was fixed for 15–30 min at room temperature or overnight at 4 °C in 4% paraformaldehyde. The tissue was then dehydrated overnight at 4 °C in 30% sucrose solution. The samples were overlaid with Optimal Cutting Temperature compound (Thermo Fisher Scientific, Waltham, MA, USA) and frozen at − 80 °C. Sections were cut at a thickness of 6–10 clip that were permeabilized for 30 min in 0.2% Triton X-100 (Sigma-Aldrich) and blocked for 1 h in 5% BSA at room temperature. The sections were then incubated overnight at 4 °C with primary antibodies, washed 3 times with PBS, incubated with secondary antibodies at room temperature for 1 h, washed 3 times with PBS, and counterstained for 5 min with 4′,6-diamidino-2-phenylindole (Sigma-Aldrich; cat. no. D9542), before imaging with a confocal microscope (LSM 880; Carl Zeiss, Jena, Germany). Antibodies used are listed in Additional file 2: Table S2.

For histological analysis, lung tissue was fixed overnight at 4 °C in 4% paraformaldehyde, dehydrated in a tissue processor (TP1020; Leica, Wetzlar, Germany), and embedded in paraffin. Sections were cut at a thickness of 5–10 μm on a microtome (Leica). Hematoxylin and eosin and Masson’s trichrome staining was performed according to standard protocols, followed by imaging with a light microscope (Nikon, Tokyo, Japan).

### Kidney capsule transplantation

Immunodeficient B-NSG (NOD-Prkdc^scid^ IL2rg^tm1^/Bcgen) mice were purchased from Beijing Biocytogen (Beijing, China) and were housed in the SPF animal facility. Organoids were transplanted under the kidney capsule of the mice. Briefly, female mice aged 6 to 9 weeks were anesthetized with isoflurane, and the fur of the left back was removed using fur clippers. Ophthalmic ointment was placed on the eyes to prevent drying of the cornea, and carprofen (5 mg/kg) was injected subcutaneously to relieve pain. Then, the left back was sterilized using chlorhexidine and isopropyl alcohol. The kidney was exposed through a left lateral incision and a small incision was made by syringe in the capsule over the caudal-lateral aspect of the kidney. Day 21 LP organoids (2–3 drops of organoids per mouse) were placed under the kidney capsule using P20 pipette. The kidney was returned to the abdomen and the incision in the abdominal wall was closed with a 5-0 absorbable suture and the skin was closed with 3 M Vetbond™ Tissue Adhesive (cat. no. 1469SB). Erythromycin was applied to the wound and the mice were placed under incandescent lamp (50–75 watt) until ambulatory. Incisions were checked daily to ensure that they were intact and not infected until they were healed. Five months after transplantation, mice were sacrificed by cervical dislocation under anesthesia and kidneys were harvested through a left lateral incision. The xenografts were fixed in 4% paraformaldehyde for further immunofluorescence and histological analyses. All experiments involving mice were approved by the Institutional Animal Care and Use Committee of Southern Medical University (IACUC approval number: L2019018).

### Bleomycin injury and in vivo orthotopic transplantation

For bleomycin injury, 1 week before transplantation, B-NSG mouse was anesthetized with pentobarbital and placed on a home-made sterilized foam plate. The mouse was hanged by its incisors on the wire and restrained with a piece of ribbon in a supine position. Ophthalmic ointment was placed on the eyes and the tongue was pulled out to the side to prevent choking. The fur of the neck was removed using fur clippers and the surgical area was sterilized using chlorhexidine and isopropyl alcohol. A skin incision (5–7 mm) along the tracheal was made by fine scissors and the tracheal cartilage was exposed by blunt dissection of subcutaneous tissue and muscle; 1 U/kg bleomycin was filled into a 1-mL syringe and was injected gently into the trachea (20 μL per mouse). The mouse was held upright for a few seconds to allow bleomycin to be inhaled into the lung. The skin was closed with 3 M Vetbond™ Tissue Adhesive, and the mouse was placed under incandescent lamp (50–75 watt) until ambulatory. On the day of transplantation, organoids were digested into single cells as described above; the cells were diluted to a concentration of 100,000/20 μL in sterile DPBS and administered to anesthetized mice via intratracheal injection following the same protocol as bleomycin injury. Five months after transplantation, mice were anesthetized to collect arterial blood and then sacrificed by cervical dislocation under anesthesia and lungs were harvested for further immunofluorescence and histological analyses.

### Arterial blood gas measurements

Mice were anesthetized with pentobarbital and placed in a supine position. Carprofen (5 mg/kg) was injected subcutaneously to relieve pain. Incision of the abdominal wall along the midline was started below the xiphoid process, continued to near the pubic bone and then along the flank to expose the viscera. The intestine was moved to the left side of the mouse and the connective tissues were removed to expose the caudal vena cava and the abdominal aorta. Arterial blood was collected from the abdominal aorta into a 1 mL syringe containing 60 IU of dry, electrolyte-balanced heparin (Sigma-Aldrich; cat. no. H3149). The partial oxygen pressure, partial carbon dioxide pressure, and oxygen saturation were measured with a blood gas and chemistry analyzer (i15 vet; EDAN Instruments, Shenzhen, China). Mice were sacrificed immediately by cervical dislocation under anesthesia and lungs were harvested for further analysis.

### Statistical analysis

Statistical analysis was performed using Prism 8 software (GraphPad, La Jolla, CA, USA). The 2-tailed Student’s t test was used to assess the statistical significance (P < 0.05) of differences between 2 experimental groups.

## Results

### Generation of SOX9^−/−^ hESCs and LP organoids

**Fig. 1:**
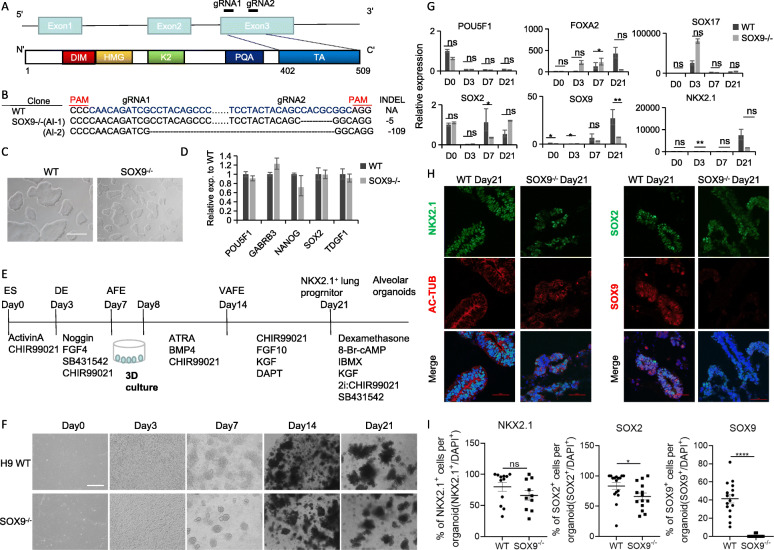
Generation of SOX9^−/−^ hESCs and differentiation of human lung organoids. **A** Schematic structures of the *SOX9* gene and protein. gRNA sites (gRNA1 and gRNA2) are indicated. DIM, dimerization domain; HMG, high-mobility group domain; K2/PQA/TA, transactivation domains. **B** Sequenced genotypes of *SOX9* wild-type (WT) and knockout (*SOX9*^*−/−*^) clones. The targeted mutation in each allele (Al-1 and Al-2) is indicated. The protospacer adjacent motif (PAM) is shown in red and gRNA sequences are shown in blue. Deletions in *SOX9* are indicated on the right side. **C** Morphology of SOX9 wild-type (WT) and knockout (SOX9^−/−^) hESCs. Scale bars, 500 μm. **D** mRNA expression levels of pluripotency-related genes. **E** Schematic of the directed differentiation protocol for generating alveolar organoids from hESCs. **F** Representative bright-field images of the differentiation time course of WT and SOX9^−/−^ cell lines. Scale bars, 500 μm. **G** mRNA expression levels of differentiation markers (n = 3, representative of 3 separate experiments). *p < 0.05, **p < 0.01 (unpaired 2-tailed Student’s t test). *POU5F1*, embryonic stem cell marker; *FOXA2* and *SOX17*, definitive endoderm markers; *NKX2.1*, lung progenitor marker; *SOX2*, embryonic stem cell and proximal airway progenitor marker; *SOX9*, distal progenitor marker. **H** Immunofluorescence labeling of NKX2.1, SOX2, SOX9, and Ac-TUB in day 21 WT and SOX9^−/−^ organoids. Scale bars, 50 μm. **I** Quantification of NKX2.1, SOX2 and SOX9 positive cells in **H**. NKX2.1: n = 12 (WT), n = 10 (SOX9^-/-^); SOX2, SOX9: n = 16 (WT), n = 14 (SOX9-/-). *p < 0.05, ****p < 0.0001 (unpaired 2-tailed Student’s t test).

Truncations or frameshift mutations in the C-terminal transactivation domain of *SOX9* have been linked to the development of CD [13]. Specifically, the C-terminal 44 residues were shown to be critical for maximal transactivation [14]. Using clustered regularly interspaced short palindromic repeats (CRISPR)/CRISPR-associated protein 9 (Cas9) technology, we designed a pair of guide RNAs (gRNAs) for targeted inactivation of the C-terminal transactivation domain of *SOX9* (Fig. 1A). We successfully generated transactivation domain knockout clonal lines (hereafter referred to as *SOX9*^-/-^) and DNA and cDNA mutations were detected by Sanger sequencing (Fig. 1B). The cell line showed typical pluripotent stem cell (PSC) morphology (Fig. 1C), and quantitative PCR (qPCR) analysis indicated that the expression levels of the pluripotency markers POU class 5 homeobox 1 (*POU5F1*), gamma-aminobutyric acid type A receptor subunit beta 3 (*GABRB3*), *NANOG*, *SOX2*, and teratocarcinoma-derived growth factor 1 (*TDGF1*) were similar to those in the parental H9 human embryonic stem cell line (hereafter referred to as wild type [WT]) (Fig. 1D).

To induce the differentiation of hESCs into LP organoids, we modified the lung differentiation protocol used in our previous work [11, 15]. (Fig. 1E). We first differentiated the hESCs into definitive endoderm (DE), and then into anterior foregut endoderm (AFE) in 2D culture. The cells were then enveloped in Matrigel to generate “ventralized” AFE (VAFE) and finally, NKX2.1-positive LPs in 3D organoids over the next 14 days (Fig. 1E, F). The qPCR analysis indicated that the pluripotency marker *POU5F1* was downregulated after 3 days; the DE markers *SOX17* and Forkhead box A2 (*FOXA2*) were upregulated on day 3 or 7; and the LP marker *NKX2.1*, proximal progenitor marker *SOX2*, and distal progenitor marker *SOX9* were upregulated on day 21 (Fig. 1G). Immunofluorescence labeling confirmed that day 21 organoids expressed NKX2.1 and SOX2. However, both qPCR and immunofluorescence analysis revealed that LP organoids derived from SOX9^−/−^ hESCs had far fewer SOX9-expressing cells than WT cells (Fig.1G–I). These results suggest that we successfully generated SOX9^-/-^ hESCs and differentiated it into lung progenitor organoids.

### Maturation of distal lung epithelial cells is not significantly affected by SOX9 inactivation

**Fig. 2:**
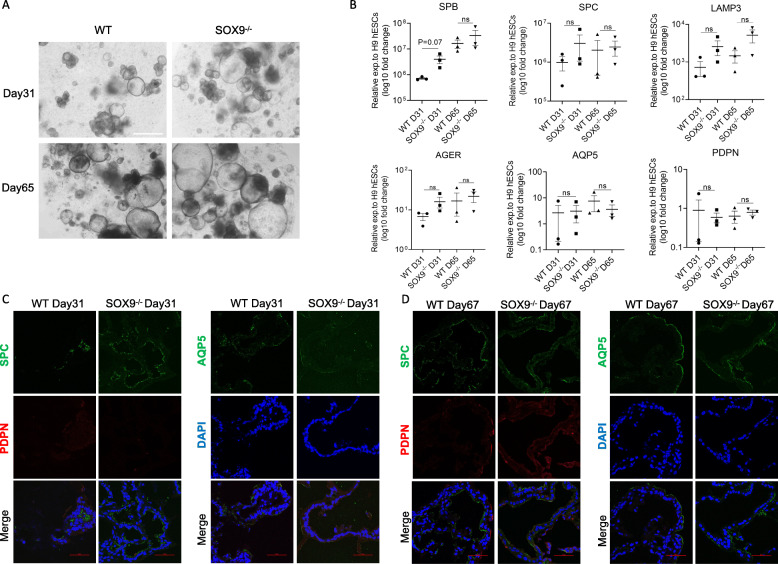
SOX9^−/−^ does not affect the maturation of AT1 and AT2 cells. **A** Representative bright-field images of SOX9 wild-type (WT) and knockout (SOX9^−/−^) organoids on day 31 or 65. Scale bars, 500 μm. **B** mRNA expression levels of AT2 markers (*SP-B*, *SP-C*, and *LAMP3*) and AT1 marker (*AGER, AQP5, PDPN*) in organoids on days 31 and 65 relative to H9 hESCs. Data represent mean ± SEM (n = 3 independent experiments). **C**, **D** Immunofluorescence labeling of AT2 markers (SP-C) and AT1 markers (PDPN and AQP5) in organoids on day 31 (**C**) and day 67 (**D**). Scale bars, 50 μm

To investigate whether SOX9 is essential for the formation of the 2 types of distal lung epithelial cell—i.e., alveolar epithelial type 2 and type 1 cells (AT2 and AT1, respectively), we further differentiated day 21 LP organoids into alveolar organoids (Fig. 1E). Bright-field microscopy analysis revealed alveolar organoids with a bubble-like structure (Fig. 2A) that expressed AT2 markers (surfactant protein B [SP-B], SP-C, and lysosomal-associated membrane protein 3 [LAMP3]) and AT1 markers (Advanced Glycosylation End-Product Specific Receptor [*AGER*], aquaporin 5 [AQP5]) after a short induction time of 31 days, as detected by qPCR. The expression of these genes was further increased after 65 days compared to day 31 organoids (Fig. 2B). Immunolabeling demonstrated that the AT2 marker SP-C and AT1 marker AQP5 were expressed in both WT and SOX9^−/−^ organoids on days 31 and 67 (Fig. 2C, D). Another AT1 marker podoplanin (PDPN) was detected on day 67 organoids (Fig. 2D). These results indicate that SOX9 inactivation does not significantly affect the maturation of alveolar organoids.

### Maturation of airway epithelial cells is not significantly affected by SOX9 inactivation

**Fig. 3:**
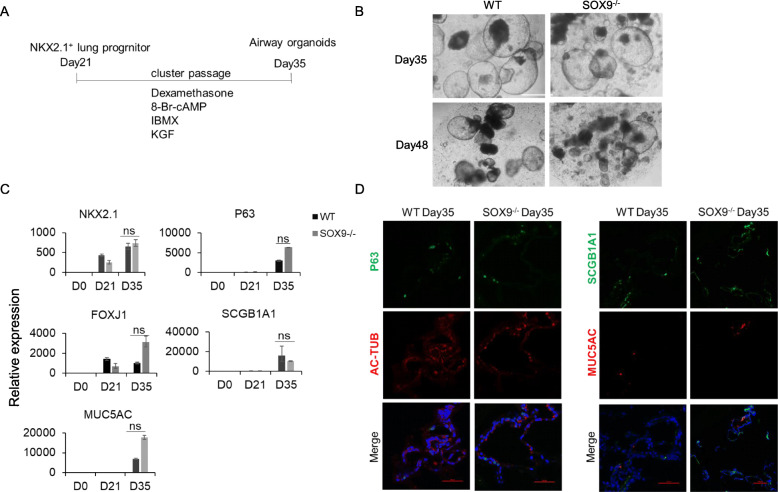
SOX9^−/−^ does not affect the maturation of airway organoids. **A** Schematic of the protocol for differentiation of organoids from day 21 NKX2.1^+^ lung progenitors. **B** Bright-field images of SOX9 wild-type (WT) and knockout (SOX9^−/−^) airway organoids on days 35 and 48. Scale bars, 500 μm. **C** mRNA expression levels of differentiation markers (n = 3, representative of 3 separate experiments). *NKX2.1*, LP marker; *P63*, basal cell marker; *FOXJ1*, ciliated cell marker; *SCGB1A1*, club cell marker; *MUC5AC*, goblet cell marker. **D** Immunofluorescence labeling of airway markers in organoids on day 35. Scale bars, 500 μm (left); 500 μm (right, WT); 100 μm (right, SOX9^−/−^)

As *SOX9* knockout in the tracheal mesenchyme resulted in aberrant differentiation of the tracheal airway epithelium in mouse [4], we examined whether it is also affected in human lung. We adjusted the alveolar organoid differentiation protocol by removing CHIR99021 (a canonical Wnt agonist that acts by inhibiting glycogen synthase kinase 3β [GSK-3β]) and SB431542 (a transforming growth factor β [TGF-β] inhibitor) after induction on day 21 (Fig. 3A). Bright-field microscopy, qPCR, and immunocytochemical analyses showed that both WT and SOX9^−/−^ airway organoids were generated by day 35 that expressed the basal cell marker P63, club cell marker secretoglobin family 1A member 1 (SCGB1A1), ciliated cell marker acetylated α-tubulin (Ac-TUB), and goblet cell marker mucin 5 AC (MUC5AC) (Fig. 3B–D). Thus, SOX9 inactivation does not affect the maturation of airway organoids.

### SOX9 inactivation reduces the proliferation and promotes apoptosis of lung organoids

**Fig. 4:**
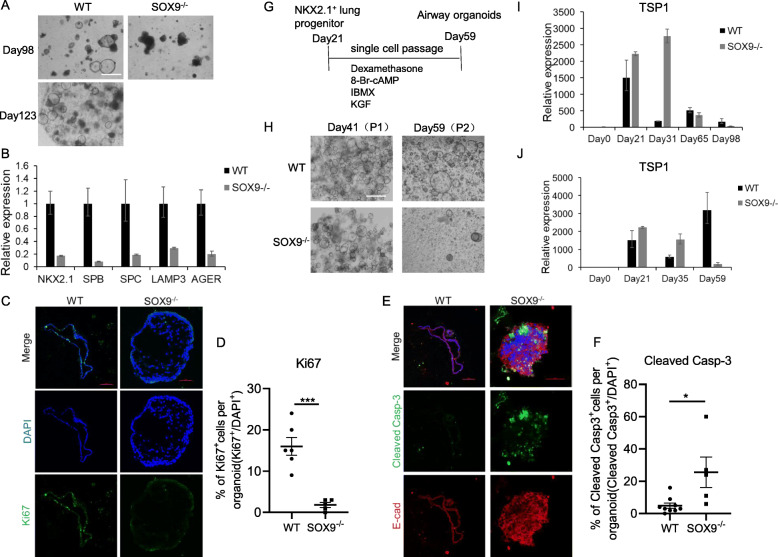
SOX9^−/−^ reduces proliferation and promotes apoptosis in lung organoids. **A** Bright-field images of day 98 SOX9 wild-type (WT) and knockout (SOX9^−/−^) alveolar organoids and day 123 WT alveolar organoids. Scale bars, 500 μm. **B** mRNA expression levels of markers in day 98 WT and SOX9^−/−^ alveolar organoids (n = 3, representative of 3 separate experiments). **C** Immunofluorescence labeling of cell proliferation marker Ki67 (green) in day 98 WT and SOX9^−/−^ alveolar organoids. Scale bars, 100 μm (left); 50 μm (right). **D** Number of Ki67-positive cells in day 98 WT (n = 6) and SOX9^−/−^ (n = 5) alveolar organoids. ***p < 0.001 (unpaired 2-tailed Student’s t test). **E** Immunofluorescence labeling of apoptosis marker cleaved caspase-3 (green) and epithelial marker E-cadherin in day 98 WT and SOX9^−/−^ alveolar organoids. Scale bars, 100 μm (left); 50 μm (right). **F** Number of cleaved caspase-3–positive cells in day 98 WT (n = 9) and SOX9^−/−^ (n = 5) alveolar organoids. *p < 0.05 (unpaired 2-tailed Student’s t test). **G** Schematic of the protocol for single-cell passaging of day 21 NKX2.1^+^ lung progenitors. (H) Bright-field images of day 41 (single cell passaged once [P1]) and day 59 (single cell passage twice [P2]) airway organoids. Scale bars, 500 μm. **I**, **J** mRNA expression levels of TSP1 during alveolar organoid (**I**) or airway organoid (**J**) differentiation (n = 3)

On day 98 of alveolar organoid differentiation, SOX9^−/−^ organoids were unhealthy as almost all organoids were died whereas WT organoids were morphologically normal and could be expanded for over 120 days (Fig. 4A). The expression levels of lung lineage markers in SOX9^−/−^ organoids were 10–20% of those observed in WT organoids, as determined by qPCR (Fig. 4B). Ki67 immunolabeling showed that the ratio of Ki67^+^ cells to total cells was significantly reduced in SOX9^−/−^ organoids compared to WT organoids, whereas the percentage of cleaved caspase-3^+^ cells was higher (Fig. 4C–F). These data suggest that SOX9 inactivation reduced proliferation and promoted apoptosis in organoids over long-term expansion.

To further confirm the effect of SOX9 inactivation on airway organoid proliferation, the organoids were passaged by digestion into single cells and repackaging into new Matrigel (Fig. 4G). After 2 generations of single-cell passaging, SOX9^−/−^ organoids did not grow well whereas WT organoids showed normal growth (Fig. 4H), indicating that SOX9 inactivation reduced the proliferative capacity of lung organoids.

It was reported that thrombospondin-1(TSP1) is a negative regulator of pluripotency transcription factors OSKM (Oct4, Sox2, Klf4, and c-Myc) [16]. In the lung, TSP1 promotes lung stem cell proliferation and differentiation [17, 18]. We therefore quantified the expression of TSP1 during lung organoids differentiation by qPCR. As expected, in ES stage (day 0) when the OSKM were highly expressed, the expression of TSP1 was nearly undetectable. Furthermore, we found the expression of TSP1 was upregulated during organoid differentiation and then downregulated in long-term culture. After long-term expansion or single-cell passaging (day 98 in alveolar organoids and day 59 in airway organoids), the expression level of TSP1 was lower in SOX9^-/-^ than WT organoids (Fig. 4I, J). These phenomena were in line with the expression pattern of some differentiation marker such as SPB, SPC, LAMP3 and AGER (Fig. 2B; Fig. 4B). Collectively, we hypothesized that inactivation of SOX9 may partly disrupt the role of TSP1 in regulating lung stem cell proliferation.

### In vivo long-term engraftment of lung organoids

**Fig. 5:**
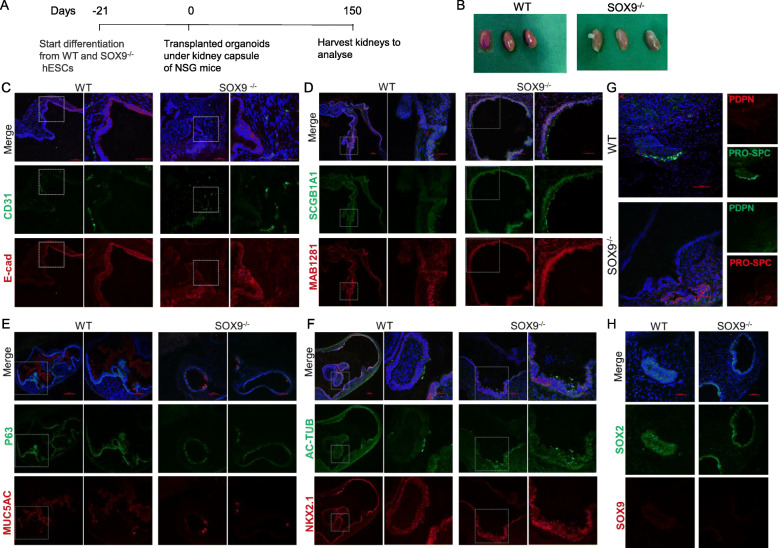
SOX9^−/−^ promotes the emergence of SCGB1A1^+^ club cells and inhibits the maturation of MUC5AC^+^ goblet cells after long-term engraftment under kidney capsule. **A** Schematic of the experimental design. **B** Images of organoids 5 months after transplantation. **C**–**H** Immunofluorescence labeling of vascular marker CD31, epithelial marker E-cad and lung lineage markers in *SOX9* wild-type (WT) and knockout (*SOX9*^*−/−*^) organoids transplanted under kidney capsule. Scale bars, 50 μm (**C**, **D**, and **F**, right panels of each sample; **E**, right panel of WT); 100 μm (**C**, **D**, and **F**, left panels of each sample; **E**, SOX9^−/−^ and left panel of WT; **G**; **H**).

We investigated the differentiation potential of WT and SOX9^−/−^ organoids by transplanting day 21 organoids—which were mainly composed of NKX2.1^+^ LPs (Fig. 1H)—under the kidney capsule of 6- to 8-week-old mice (Fig. 5A). Five of the 6 organoids survived for more than 5 months (Fig. 5B). We next examined the angiogenic activity of the grafts and found that there were CD31^+^ endothelial cells forming microvessels in both WT and SOX9^-/-^ grafts (Fig. 5C). Immunolabeling revealed that both WT and SOX9^−/−^ grafts expressed the human nuclear marker MAB1281, as well as the LP marker NKX2.1, proximal stem cell marker SOX2, basal cell marker P63, ciliated cell marker Ac-TUB, and goblet cell marker MUC5AC (Fig.5D-F). Notably, a group of cells in SOX9^−/−^ transplants expressed the club cell marker SCGB1A1, which was absent in WT grafts (Fig. 5D). This is similar to the observation in mouse that Sox9 inactivation in mesenchyme resulted in more cells in the trachea that were positive for Scgb1a1 [4]. Additionally, there were differences between the 2 grafts in terms of the localization of MUC5AC: in the WT graft, most of the protein was secreted into the lumen, whereas in SOX9^−/−^ grafts it remained in the cytoplasm (Fig. 5E). Both WT and SOX9^−/−^ grafts expressed the AT2 marker pro–SP-C but lacked the distal stem cell marker SOX9 and AT1 marker PDPN (Fig. 5G, H). These data demonstrate that when transplanted under kidney capsule, both WT and SOX9^−/−^ organoids mature, but with some differences.

**Fig. 6:**
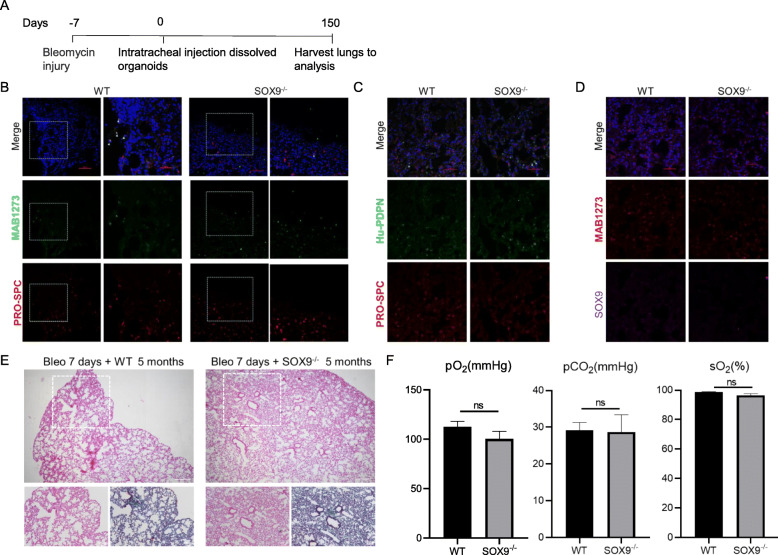
SOX9^−/−^ does not affect the differentiation of alveolar cells or their role in promoting the functional recovery of injured mice. **A** Schematic of the experimental design. **B**–**D** Immunofluorescence labeling of human mitochondria marker MAB1273, AT2 marker pro–SP-C, AT1 marker human PDPN (HU-PDPN), and distal progenitor marker SOX9 in transplanted SOX9 wild-type (WT) and knockout (SOX9^−/−^) lung progenitor organoids. White arrowhead in **B** indicates cells coexpressing MAB1273 and pro–SP-C; white arrowhead in **C** indicates cells coexpressing HU-PDPN and pro–SP-C. n = 4 (WT), n = 5 (SOX9^-/-^). Scale bars, 100 μm (**B**, left panels of each sample); 50 μm (**B**, right panels of each sample; **C**; **D**). **E** Hematoxylin and eosin (H&E) and Masson’s trichrome staining of transplanted WT and SOX9^−/−^ lung progenitor organoids. Scale bars, 500 μm (top); 100 μm (bottom). **F** Arterial blood gas analysis. Partial oxygen pressure (pO_2_), partial carbon dioxide pressure (pCO_2_), and oxygen saturation (sO_2_) were measured in WT and SOX9^−/−^ organoid-transplanted mice. Data represent mean ± SEM (n = 3)

As SOX9 is a distal progenitor marker of the lung, we examined whether inactivation of SOX9 affects the maturation of distal lung epithelium. Damage to the lung epithelium has been shown to improve graft survival, and hPSC-derived lung bud tip progenitor cells survived for up to 6 weeks in NOD/SCID/IL2rγ^null^ (NSG) mice while giving rise to multiple epithelial cell lineages [19]. We therefore used a bleomycin-induced alveolar damage model to determine whether SOX9 inactivation affected the ability of the cells to regenerate lungs (Fig. 6A). Immunofluorescence analysis showed that in both WT and SOX9^−/−^ transplanted lungs, a small proportion of cells were positive for the human mitochondria marker MAB1273 (Fig. 6B, D). Some cells co-expressed MAB1273 and pro–SP-C, indicating a potential to differentiate into AT2 cells (Fig. 6B). Meanwhile, most of the engrafted cells were positive for human PDPN expression, implying that they were AT1 cells (Fig. 6C). Only a few cells were in a PDPN^+^/SP-C^+^ bipotent progenitor state and none were MAB1273^+^/SOX9^+^, suggesting that nearly all transplanted cells were undergoing maturation (Fig. 6C, D). Hematoxylin and eosin and Masson’s trichrome staining revealed areas of fibrosis reflecting damage caused by bleomycin in both WT and SOX9^−/−^ transplanted mice (Fig. 6E). Pulmonary function was improved in both groups with no significant difference between them (Fig. 6F). Taken together, our results demonstrate that inactivation of SOX9 does not affect the differentiation of alveolar cells or their ability to promote functional recovery of injured lung in mice.

## Discussion

In this study, we used CRISPR/Cas9-mediated gene editing to inactivate SOX9 in hESCs. SOX9^−/−^ hESCs could be induced to form lung organoids, suggesting that SOX9 is not a key transcription factor regulating the fate decision of human LPs. Using hPSC-derived lung organoids, we studied the role of SOX9 in human lung epithelium development for the first time. Mutations in *SOX9* were identified as the cause of campomelic dysplasia (CD) in humans [3, 20]. Infants born with CD could not survive the neonatal period because of respiratory distress [21]. We therefore believe that the method we took and the phenotypes we found might help to understand the mechanism of respiratory distress in CD patients.

In mouse models, Sox9 inactivation resulted in the early differentiation of lung alveoli, with elevated expression of AT2 genes such as *Sftpc* and *Sftpb* (encoding SP-C and SP-B, respectively) and *Lamp3* [6, 22]. Although our results showed that transcript levels were slightly elevated in SOX9^−/−^ alveolar organoids relative to WT organoids, there were no statistically significant differences in either mRNA or protein expression (Fig. 2B–D). As immune defense-related factors (SP-A and SP-D) and AT1 markers (receptor for advanced glycation end products [RAGE] and AQP1) are unaffected by Sox9 mutation [22], we speculate that the reason alveolar differentiation was not greatly affected in SOX9^−/−^ organoids is due to interspecies differences or because this process is controlled by SOX9-independent mechanisms [22]. In vivo orthotopic transplantation results confirmed that SOX9 inactivation did not affect AT1 and AT2 cell differentiation or their role in tissue repair after lung damage (Fig. 6).

Inactivation of Sox9 in lung mesenchyme was found to alter the tracheal epithelium in mouse, with more Scgb1a1^+^ club cells and fewer P63^+^ basal cells and AGR2^+^ goblet cells [4]. In the present study, the tracheal epithelium appeared normal after SOX9 inactivation in airway organoids (Fig. 3). However, our kidney transplantation experiment showed that SOX9 inactivation led to the emergence of SCGB1A1^+^ club cells and inhibited the maturation of MUC5AC^+^ goblet cells (Fig. 5), which is consistent with earlier findings [4].

A key finding of our work is that SOX9 inactivation affected the proliferative capacity of lung organoids (Fig. 4). It was previously reported that loss of Sox9 reduced proliferation in the distal epithelium but had no effect in the proximal epithelium [6], but in our study, both proximal and distal organoids were affected. This implies that SOX9 is involved in the maintenance of lung stem cells and that its inactivation promotes their apoptosis.

Ultimately, our findings, together with previously published works [10, 23, 24], have proved that hPSC-derived lung organoids are powerful tools to model human lung development and diseases. However, lung organogenesis is regulated by multiple factors, including complex mesenchymal-epithelial interactions, extracellular matrix (ECM) remodeling, and physical forces such as fluid pressure and the basal degree of lung expansion [25, 26]. Alterations to any of these factors could have dramatic and long-term impacts on the lung. To date, lung organoids cultured in vitro cannot achieve full maturation to the adult stage [27–29]. After 6 months of culture, the organoids only matched the second trimester of human gestation [27, 28]. Even with technical modification, only a fraction of the cells was able to undergo further maturation [29]. The absence of cell diversity (e. g., endothelial and immune cells), naïve ECM components, and physiological-like mechanical stress in organoids are the limitations [30]. Further optimization of the lung organoids is needed to make them as faithful models of human biology.

## Conclusions

In this study, we used CRISPR/Cas9 technology and hESC-derived lung organoids to demonstrate that SOX9 affects the proliferative capacity of lung epithelial cells but may not be an indispensable transcription factor regulating the development of human lung epithelium. Using this model, it is possible to study the embryonic development of human organs, which was previously only approximated by animal models such as zebrafish or mouse.

## Supplementary Information

**Additional file 1.**

**Additional file 2.**

## Data Availability

The data that support the findings of this study are available from the corresponding author upon reasonable request.

## Abbreviations

LPs: Lung progenitors
NKX2.1: NK2 homeobox 1
SOX2: Sex-determining region Y-box transcription factor 2
SOX9: Sex-determining region Y-box transcription factor 9
Id2: Inhibitor of DNA-binding 2
CD: Campomelic dysplasia
3D: 3-Dimensional
hESCs: Human embryonic stem cells
CRISPR/Cas9: Clustered regularly interspaced short palindromic repeats (CRISPR)/CRISPR-associated protein 9 (Cas9)
gRNAs: Guide RNAs
PSC: Pluripotent stem cell
POU5F1: Pluripotency markers POU class 5 homeobox 1
GABRB3: Gamma-aminobutyric acid type A receptor subunit beta 3
NANOG: Nanog Homeobox
TDGF1: Teratocarcinoma-derived growth factor 1
DE: Definitive endoderm
AFE: Anterior foregut endoderm
VAFE: “Ventralized” anterior foregut endoderm
WT: Wild type
FOXA2: Forkhead box A2
AT1: Alveolar epithelial type 1
AT2: Alveolar epithelial type 2
SPB: Surfactant protein B
SPC: Surfactant protein C
LAMP3: Lysosomal-associated membrane protein 3
AGER: Advanced Glycosylation End-Product Specific Receptor
AQP5: Aquaporin 5
SCGB1A1: Secretoglobin family 1A member 1
Ac-TUB: Acetylated α-tubulin
MUC5AC: Mucin 5 AC
PDPN: Podoplanin
TSP1: Thrombospondin-1
NSG: NOD/SCID/IL2rγ^null^
RAGE: Receptor for advanced glycation end products
ECM: Extracellular matrix

## References

[1] Herriges M, Morrisey EE (2014). Lung development: orchestrating the generation and regeneration of a complex organ. Development..

[2] Nikolić MZ, Caritg O, Jeng Q, Johnson JA, Sun D, Howell KJ, et al. Human embryonic lung epithelial tips are multipotent progenitors that can be expanded in vitro as long-term self-renewing organoids. Elife. 2017;6. 10.7554/eLife.26575. 10.7554/eLife.26575 PMC5555721 28665271

[3] Wagner T, Wirth J, Meyer J, Zabel B, Held M, Zimmer J, Pasantes J, Bricarelli FD, Keutel J, Hustert E, Wolf U, Tommerup N, Schempp W, Scherer G (1994). Autosomal sex reversal and campomelic dysplasia are caused by mutations in and around the SRY-related gene SOX9. Cell..

[4] Turcatel G, Rubin N, Menke DB, Martin G, Shi W, Warburton D (2013). Lung mesenchymal expression of Sox9 plays a critical role in tracheal development. BMC Biol..

[5] Perl AK, Kist R, Shan Z (2005). Normal lung development and function after Sox9 inactivation in the respiratory epithelium. Genesis..

[6] Rockich BE, Hrycaj SM, Shih HP, Nagy MS, Ferguson MAH, Kopp JL, Sander M, Wellik DM, Spence JR (2013). Sox9 plays multiple roles in the lung epithelium during branching morphogenesis. Proc Natl Acad Sci U S A..

[7] Turcatel G, Millette K, Thornton M, Leguizamon S, Grubbs B, Shi W, Warburton D (2017). Cartilage rings contribute to the proper embryonic tracheal epithelial differentiation, metabolism, and expression of inflammatory genes. Am J Physiol Lung Cell Mol Physiol..

[8] Jo A, Denduluri S, Zhang B, Wang Z, Yin L, Yan Z, Kang R, Shi LL, Mok J, Lee MJ, Haydon RC (2014). The versatile functions of Sox9 in development, stem cells, and human diseases. Genes Dis..

[9] Li M, Izpisua Belmonte JC (2019). Organoids – preclinical models of human disease. N Engl J Med..

[10] Lancaster MA, Knoblich JA (2014). Organogenesis in a dish: modeling development and disease using organoid technologies. Science..

[11] Yamamoto Y, Gotoh S, Korogi Y, Seki M, Konishi S, Ikeo S, Sone N, Nagasaki T, Matsumoto H, Muro S, Ito I, Hirai T, Kohno T, Suzuki Y, Mishima M (2017). Long-term expansion of alveolar stem cells derived from human iPS cells in organoids. Nat Methods..

[12] Dye BR, Hill DR, Ferguson MA, et al. In vitro generation of human pluripotent stem cell derived lung organoids. Elife. 2015;4. 10.7554/eLife.05098. 10.7554/eLife.05098 PMC4370217 25803487

[13] McDowall S, Argentaro A, Ranganathan S, Weller P, Mertin S, Mansour S, Tolmie J, Harley V (1999). Functional and structural studies of wild type SOX9 and mutations causing campomelic dysplasia. J Biol Chem..

[14] Südbeck P, Schmitz ML, Baeuerle PA, Scherer G (1996). Sex reversal by loss of the C-terminal transactivation domain of human SOX9. Nat Genet..

[15] Chen Y, Feng J, Zhao S, Han L, Yang H, Lin Y, Rong Z (2018). Long-term engraftment promotes differentiation of alveolar cells from human embryonic stem cell derived lung organoids. Stem Cells Dev..

[16] Kaur S, Soto-Pantoja DR, Stein EV, Liu C, Elkahloun AG, Pendrak ML, Nicolae A, Singh SP, Nie Z, Levens D, Isenberg JS, Roberts DD (2013). Thrombospondin-1 signaling through CD47 inhibits self-renewal by regulating c-Myc and other stem cell transcription factors. Scientific reports..

[17] Lee JH, Bhang DH, Beede A, Huang TL, Stripp BR, Bloch KD, Wagers AJ, Tseng YH, Ryeom S, Kim CF (2014). Lung stem cell differentiation in mice directed by endothelial cells via a BMP4-NFATc1-thrombospondin-1 axis. Cell..

[18] Li K, Wu Q, Sun X, Geng Y, Leng D, Li H, Zhang S, Wang Q, Wu J, Xu L, Li X, Li Y, Zhang Q, Kurkciyan A, Liang J, Jiang D, Chen H (2017). Tsp1 promotes alveolar stem cell proliferation and its down-regulation relates to lung inflammation in intralobar pulmonary sequestration. Oncotarget..

[19] Miller AJ, Hill DR, Nagy MS, Aoki Y, Dye BR, Chin AM, Huang S, Zhu F, White ES, Lama V, Spence JR (2018). In vitro induction and in vivo engraftment of lung bud tip progenitor cells derived from human pluripotent stem cells. Stem Cell Reports..

[20] Kwok C, Weller PA, Guioli S, Foster JW, Mansour S, Zuffardi O, Punnett HH, Dominguez-Steglich MA, Brook JD, Young ID (1995). Mutations in SOX9, the gene responsible for Campomelic dysplasia and autosomal sex reversal. Am J Human Genet..

[21] Mansour S, Hall CM, Pembrey ME, Young ID (1995). A clinical and genetic study of campomelic dysplasia. Journal of medical genetics..

[22] Chang DR, Martinez Alanis D, Miller RK, Ji H, Akiyama H, McCrea PD, Chen J (2013). Lung epithelial branching program antagonizes alveolar differentiation. Proc Natl Acad Sci U S A..

[23] McCauley KB, Hawkins F, Serra M (2017). Efficient derivation of functional human airway epithelium from pluripotent stem cells via temporal regulation of Wnt signaling. Cell stem cell..

[24] Jacob A, Morley M, Hawkins F, McCauley KB, Jean JC, Heins H, Na CL, Weaver TE, Vedaie M, Hurley K, Hinds A, Russo SJ, Kook S, Zacharias W, Ochs M, Traber K, Quinton LJ, Crane A, Davis BR, White FV, Wambach J, Whitsett JA, Cole FS, Morrisey EE, Guttentag SH, Beers MF, Kotton DN (2017). Differentiation of human pluripotent stem cells into functional lung alveolar epithelial cells. Cell stem cell..

[25] Morrisey EE, Hogan BL (2010). Preparing for the first breath: genetic and cellular mechanisms in lung development. Developmental cell..

[26] Zepp JA, Morrisey EE (2019). Cellular crosstalk in the development and regeneration of the respiratory system. Nature reviews Molecular cell biology..

[27] Chen YW, Huang SX, de Carvalho A (2017). A three-dimensional model of human lung development and disease from pluripotent stem cells. Nature cell biology..

[28] Porotto M, Ferren M, Chen YW, Siu Y, Makhsous N, Rima B, et al. Authentic modeling of human respiratory virus infection in human pluripotent stem cell-derived lung organoids. mBio. 2019;10(3):e00723–19. 10.1128/mBio.00723-19. 10.1128/mBio.00723-19 PMC6509192 31064833

[29] de Carvalho ALRT, Strikoudis A, Liu HY, Chen YW, Dantas TJ, Vallee RB, et al. Glycogen synthase kinase 3 induces multilineage maturation of human pluripotent stem cell-derived lung progenitors in 3D culture. Development. 2019;146(2):dev171652. 10.1242/dev.171652. 10.1242/dev.171652 PMC6361135 30578291

[30] Tian L, Gao J, Garcia IM, Chen HJ, Castaldi A, Chen YW. Human pluripotent stem cell-derived lung organoids: potential applications in development and disease modeling. WIREs Dev Biol. 2020;e399. 10.1002/wdev.399. 10.1002/wdev.399 33145915

